# *ADAM* and *ADAMTS* gene expression in native and wound healing human lens epithelial cells

**Published:** 2010-12-15

**Authors:** Lisa M. Hodgkinson, Lixin Wang, George Duncan, Dylan R. Edwards, I. Michael Wormstone

**Affiliations:** School of Biological Sciences, University of East Anglia, Norwich, UK

## Abstract

**Purpose:**

The ADAMs (a disintegrin and metalloproteinase) and the ADAMTSs (a disintegrin and metalloproteinase with thrombo**s**pondin-like motifs) are extracellular proteases that mediate cellular interactions and cell signaling via the modulation of adhesion and the cleavage of cell surface protein ectodomains and extracellular matrix molecules. Gene expression profiling was undertaken to better understand the role of the ADAM and ADAMTS families in the clear native human lenses and following surgical injury with particular relevance to posterior capsule opacification.

**Methods:**

To carry out profile analysis, the lens (t=0d) was dissected into three regions; anterior epithelia, equatorial region, and fiber cells. Capsular bag culture was undertaken as a means of assessing short-term changes (t=6d) and post-cataractous lens capsular bags (ex vivo) were used to predict long-term changes in *ADAM*/*ADAMTS* gene expression. RNA was isolated and quantitative real-time (TaqMan) reverse transcription-PCR (RT–PCR) performed. Data were analyzed in terms of cycle threshold number (C_T_) and also normalized relative to endogenous 18S rRNA.

**Results:**

High expression of *ADAM-9*, *-10*, *-15*, and *-17* was detected in all native lens regions. *ADAM-15* expression was a characteristic of the native lens epithelia more than the fibers. Post-surgical injury, lens capsular bags showed a positive shift in *ADAM*/*ADAMTS* expression that was significant for *ADAM-9*, *-15*, and *ADAMTS-3*. Ex vivo capsular bags, with a long-term post surgical injury period, maintained high expression of *ADAM-9* and *-10* genes.

**Conclusions:**

The native human lens expresses *ADAM* and *ADAMTS* genes that are differentially regulated following surgical injury. Roles in maintaining cellular adhesion may be of particular importance to native lens tissue integrity and may be lost in the lens wound healing response following cataract surgery.

## Introduction

The ADAMs (a disintegrin and metalloproteinase) and the ADAMTSs (a disintegrin and metalloproteinase with thrombospondin-like motifs) are members of the M12 adamalysin family of the metzincin metalloproteinases, related to the matrix metalloproteinases (MMPs) [1,2]. They are highly influential, multifunctional enzymes that regulate the extracellular microenvironment as well as cell signaling. In particular, the ADAMs have adhesive properties via their disintegrin and Cys-rich domains, while those that are active proteases mediate diverse protein ectodomain shedding events that liberate and regulate biologically active molecules at the cell surface [1,2]. The ADAMTS family members have roles in the processing of procollagen molecules and cleavage of matrix hyalectans such as aggrecan, brevican and versican, while ADAMTS-13 is involved in hemostasis as the von Willebrand factor-cleaving proteinase [3]. Several ADAM/ADAMTS enzymes have roles in cell differentiation and cell guidance mechanisms during development [2,3]. The ADAM/ADAMTSs are thus likely to be relevant to the function of the normal lens and alterations in their expression may be significant in lens pathologies, such as posterior capsule opacification (PCO) [4].

Up to 34 ADAM orthologs have now been discovered in many species from vertebrates to *Caenorhabditis elegans*, including 19 human ADAMs [5]. ADAM structure comprises a prodomain and a metalloproteinase domain followed by a disintegrin domain that has the ability to associate with integrins [2]. Linked to this are cysteine-rich and EGF-like domains that attach to a transmembrane domain and cytoplasmic tail [6,7]. The ADAMTS are structurally distinguished by the inclusion of often multiple thrombospondin-like motifs, the lack of the transmembrane domain which gives them extracellular functions, and also the lack of the EGF-like domain. Since the discovery of the first ADAMTS in 1997, 19 human ADAMTS family members have been described [8,9].

There is limited published research on the role of ADAM/ADAMTS families in the eye. In the developing chick cornea ADAM-10 has been linked with cell migration [10] and in the human cornea ADAMTS-1 interacts with fibulin proteins, which are ECM components that regulate organ shape [11]. Additionally, a study conducted during chick embryogenesis localized ADAM-35 (meltrin ε) to epithelial tissues derived from the surface ectoderm, including the lens placode and subsequently to the lens vesicles [12].

There are a small number of references that link the ADAM/ADAMTS with abnormal eye pathology. With respect to the lens; decreased ADAM-9 expression was detected in human anterior polar cataractous lenses (also referred to as anterior subcapsular cataract; ASC) relative to clear controls and ADAM-9 expression could be differentially regulated in BALB/c wild type mouse lens epithelial explants by culturing with TGFβ [13]. Additionally, lenticular abnormality in the connective tissue disorder Weill-Marchesani syndrome has been linked with *ADAMTS-10* gene mutation [14]. Using a proteomic approach, ADAM-19, −21, and ADAMTS-8 were detected in the anterior lens capsules of patients with co-existing Exfoliation syndrome (XFS) [15]. XFS is a major cause of glaucoma in which abnormal matrix deposits occur in the anterior segment (often in close relation to the lens) and is associated with cataract [16].

Limited information is available regarding gene expression in individual human lenses and therefore members of the *ADAM*/*ADAMTS* gene families were selected for study in the native lens, the wound healing lens and the post-cataractous lens capsular bag (ex vivo). The genes selected encode catalytically active ADAMs with a membrane-anchored metalloproteinase domain containing a catalytic-site consensus sequence [2]. The selected *ADAMTS* genes encode secreted ADAMTS proteins that were identified for their enzyme substrate specificities and had likely relevance to the lens. The candidate genes were *ADAM-8*, *-9*, *-10*, *-12*, *-15*, *-17*, −19, and *-28*, and the *ADAMTS* were *ADAMTS-1*, *-2*, *-3*, and *-14*.

The emphasis for this study was to report the gene expression of selected *ADAM*/*ADAMTS* in individual human lenses and so we used techniques that had been previously developed [17]. *ADAM*/*ADAMTS* gene expression patterns were analyzed in different regions of the same lens that had undergone; (1) sham cataract surgery in vitro to mimic the in vivo reality of a cataract operation (t=0), (2) in capsular bags following sham surgery in vitro that were cultured in unsupplemented medium for six days (t=6d) and in (3) capsular bags ex vivo that had previously undergone cataract surgery before death. The information gained provides insights into the potential roles of *ADAM*s/*ADAMTS*s in both the intact normal human lens and during wound healing responses following surgery.

## Methods

### Donor lens selection and short-term lens culture

Human donor eyes were obtained with full ethical permission from the East Anglian Eye Bank (EAEB) or the Corneal Transplant Service (Eye Bank, Bristol) after corneo-scleral discs had been removed for transplantation purposes. The use of human tissue in the study was in accordance with the provisions of the Declaration of Helsinki. All eyes were stored in individual sterile pots in an antibiotic wash medium before use. Regional dissection for gene expression analysis [17] and capsular bag culture methods [18] have been published previously.

The following human donor eyes were obtained postmortem for gene expression analyses: native (t=0) n=3, age range 74–84 years, mean=79.3 years; for culture (t=6d): n=3, age range 69–76, mean=73.7; and for analyses of capsular bags from donors who had previously undergone cataract surgery (ex vivo): n=3, age range 70–76, mean=73.6. In the latter case the time between surgery and death was not known, however there was evidence of PCO in all cases; notably cells had encroached upon the central posterior capsule and exhibited fibrotic changes such as matrix wrinkling and cell aggregation. Native lenses were dissected into three regions; anterior epithelia, equatorial region and fibers using published methodology [17], as mentioned. Briefly, the iris was removed and a circular anterior epithelium (capsulorhexis) torn away. The lens nucleus was then released by hydroejection and residual fibers were carefully sampled using forceps. Finally the lens capsule was dissected from the zonules.

Capsular bags for short-term culture were secured on to a sterile PMMA culture dish using six to eight entomological pins (D1; Watkins and Doncaster Ltd., Kent, UK), inserted through the edge of the capsular bag to maintain its shape and were then cultured for six days in unsupplemented Eagle minimal essential medium (EMEM; Sigma, Dorset, UK). The capsular bags from donors who had undergone cataract surgery and intra-ocular lens (IOL) implantation before death were dissected free from the globe as described and the IOL was removed. Immediately following preparation each sample was snap-frozen in liquid nitrogen.

### Total RNA extraction and cDNA generation

Total RNA was extracted from lens tissue and cultured capsular bag samples according to the manufacturers of the RNeasy micro kits (Qiagen, West Sussex, UK). In the initial step RLT buffer (containing β-mercaptoethanol) was added to Eppendorf tubes containing snap frozen samples. The samples were then homogenized using an Eppendorf homogenizer and were then passed through a 20 gauge needle (0.9 mm) and syringe. The remainder of the protocol was as described by the manufacturer and included a DNase step. Quality control was performed to ensure that 28S and 18S rRNA bands were clearly evident in total RNA samples using an Agilent Bioanalyzer 2100 (Agilent, West Lothain, UK) and a RNA 6000 Nano labchip. RNA was quantified using a NanoDrop ND-1000 spectrophotometer (NanoDrop, Wilmington, DE). For the total of 15 samples analyzed, the ratio of adsorptions at 260/280 nm ranged from 1.8 to 2.2 (mean=2.0). Where possible, total RNA was immediately used for cDNA generation or briefly stored over night at −80 °C. Generation of cDNA was performed with Superscript II reverse transcriptase (Invitrogen, Paisley, UK) according to the reverse transcription (RT) protocols of the manufacturer using random primers (Promega, Southampton, UK).

### Real time PCR

Real time PCR was used to quantitate mRNA for all genes expressed by native tissue, cultured and ex vivo capsular bags, relative to endogenous control genes. Oligonucleotide primers and fluorescence-labeled probes for *ADAM*/*ADAMTS* genes were designed in house using Primer Express 1.0 software (Applied Biosystems, Foster City, CA). The sequences are presented in Table 1. A pre-designed TaqMan® Gene expression assay was purchased (Applied Biosystems) for eukaryotic 18S rRNA expression quantitation (NCBI Reference Sequence [RefSeq] at the time of publication; X03205.1 and primer probe set ID; Hs99999901_s1) and used according to the manufacturer’s instructions.

**Table 1:** Taqman primer/probe sets for *ADAM* and *ADAMTS* family members.

**Gene**	**Reference**	**Forward primer (5’-3’)**	**Reverse primer (3’ – 5’)**	**Probe (5’-FAM – TAMRA-3’)**	**NCBI accession number**
ADAM 8	[20]	AAGCAGCCGTGCGTCATC	AACCTGTCCTGACTATTCCAAATCTC	AATCACGTGGACAAGCTATATCAGAAACTCAACTTCC	NM_001109
ADAM9	-	GGAAACTGCCTTCTTAATATTCCAAA	CCCAGCGTCCACCAACTTAT	CCTGATGAAGCCTATAGTGCTCCCTCCTGT	NM_003816.2 , NM_001005845.1
ADAM10	[19]	AGCGGCCCCGAGAGAGT	AGGAAGAACCAAGGCAAAAGC	ATCAAATGGGACACATGAGACGCTAACTGC	NM_001110
ADAM12	[20]	AGCTATGTCTTAGAACCAATGAAAAGTG	CCCCGGACGCTTTTCAG	ACCAACAGATACAAACTCTTCCCAGCGAAGA	NM_003474 , NM_021641
ADAM15	-	CCAGCTGTCACCCTCGAAA	GGCAATCGAGGCAGCAAA	TTCCTCCACTGGCGCAGGGC	NM_207191 , NM_003815, NM_207194, NM_207195, NM_207196, NM_207197
ADAM17	[19]	GAAGTGCCAGGAGGCGATTA	CGGGCACTCACTGCTATTACC	TGCTACTTGCAAAGGCGTGTCCTACTGC	NM_003183
ADAM19	-	TGACAGCAAGGGCCAACAC	AGGTCGCTTCTTGGTCTGTTGT	TCGAGCACTCCAAGCCCACCACC	NM_023038 , NM_033274
ADAM28	-	GGGCCCACGATTTGCA	TGAACCTTCCTGTCTTTCAATTTTACT	AGAACATTGCCCTACCTGCCACCAAAC	NM_014265 , NM_021777
ADAMTS1	[21]	GGACAGGTGCAAGCTCATCTG	TCTACAACCTTGGGCTGCAAA	CAAGCCAAAGGCATTGGCTACTTCTTCG	NM_006988.2
ADAMTS2	[21]	CTGGCAAGCATTGTTTTAAAGGA	GGAGCCAAACGGACTCCAA	ATCTGGCTGACACCTGACATCCTCAAACG	NM_014244.1
ADAMTS3	[21]	GCAGCATTCCATCGTTACCA	CCATAGAATAATTGATTCCAGGAAGTT	CCATTCCTATGACTGTCTCCTTGATGACCC	NM_014243.1
ADAMTS4	[21]	CGCTGGATGGGACTGAGTGT	CGCGAACATGACCCAAACTT	CCCGGCAAGTGGTGCTTCAAAGGT	AF366351.1

Assuming 100% efficiency in the RT reactions, either 1 or 5 ng cDNA was used in real-time PCR reactions performed using a real-time PCR machine (ABI7700; Applied Biosystems). Reagent-based assays (TaqMan Universal PCR Master Mix, No AmpErase^®^ UNG; Applied Biosystems) containing all PCR reagents were employed according to the manufacturer’s instructions. The amount of amplification associated with priming from genomic DNA contamination was evaluated in control RT reactions that contained all reagents and total RNA sample template without reverse transcriptase. Conditions for the PCR reaction were; 2 min at 50 °C, 10 min at 95 °C and then 40 cycles, each consisting of 15 s at 95 °C and 1 min at 60 °C. The cycle number at which amplification entered the exponential phase (raw data cycle threshold [C_T_]) was determined and this number was used as an indicator for the amount of target RNA in each lens tissue sample analyzed. In qualitative raw data analyses the C_T_ value was used to classify gene expression as either very high (C_T_≤20), high (C_T_=21–25), moderate (C_T_=26–30), low (C_T_=31–38), or negligible to undetected (C_T_=39–40). To determine the relative RNA levels in the lens tissue samples, standard curves for each primer/probe set were prepared by using cDNA from one sample and making twofold serial dilutions covering the range equivalent to 20–0.625 ng RNA (for 18S analysis the range was from 1 to 0.03125 ng). Differences in the total amount of RNA present in each sample were normalized to endogenous 18S rRNA gene expression.

### Statistical analysis

To provide multiple group comparison, statistical analysis was performed using one-way ANOVA with Tukey’s post-hoc analysis using p≤0.05 to gauge significance.

## Results

### *ADAM*/*ADAMTS* gene expression determined by C_T_ analysis in regions of the native human lens

**Figure 1:**
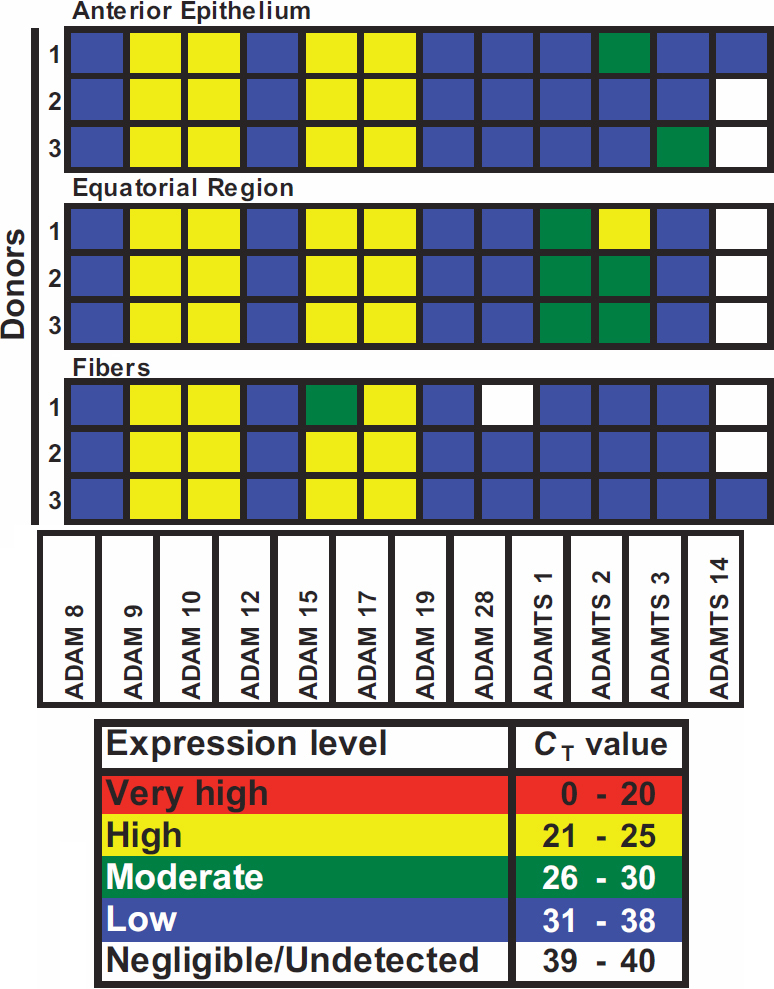
*ADAM*/*ADAMTS* gene expression profiles were classified in lens regions (donors 1–3). The cycle threshold number (C_T_) was used to classify gene expression as either very high (C_T_≤20), high (C_T_=21–25), moderate (C_T_=26–30), low (C_T_=31–38) or negligible to undetected (C_T_=39–40). Donor information; number: (1–3), sex (F; female, M; male), age and ophthalmic history; 1: F, 74, none; 2: M, 84, none; 3: M, 81, none.

An initial qualitative analysis of the raw data cycle threshold (C_T_) values before 18S rRNA normalization is presented in Figure 1, which shows the relative gene expression ranges for the selected *ADAM*/*ADAMTS* genes detected in three individual native donors at t=0. This is a useful basic analysis to compare the relative expression levels of genes in categories of undetected, low, moderate, high and very high, which we have previously validated extensively [21,22]. Using the C_T_ value as a measure, the majority of the *ADAM*/*ADAMTS* genes analyzed were expressed at the same level in all three lens regions and were classified as undetected or low, except for *ADAMs-9*, *-10*, *-15*, and *-17*. These *ADAM* family members were in all cases classified as highly expressed, except for one donor tissue that expressed moderate levels of *ADAM-15* in lens fibers. Interestingly, expression of *ADAMTS-1* and *-2* was low in the anterior epithelia and fiber cells, but was moderate to high in the equatorial region. *ADAMTS-14* was largely classified as negligible or undetected in the samples tested.

### *ADAM*/*ADAMTS* gene expression in regions of the native human lens determined using a relative standard curve

**Figure 2:**
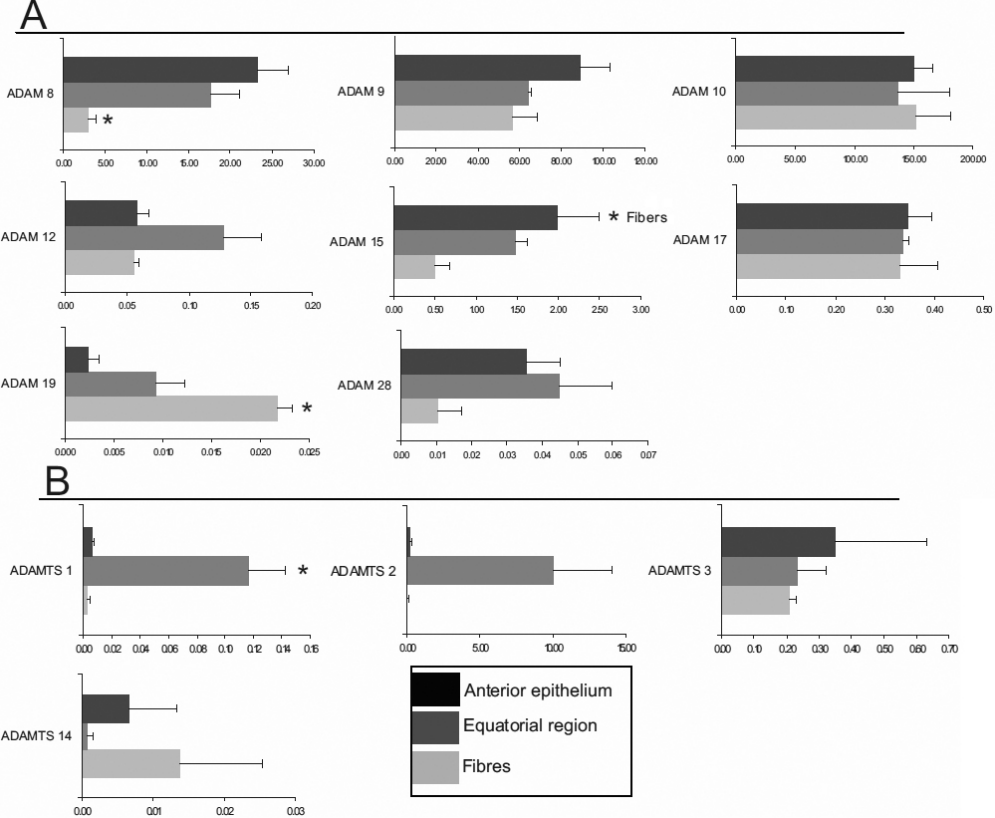
Quantitative comparison of regional *ADAM*/*ADAMTS* gene expression. **A**: *ADAM* and **B**: *ADAMTS* gene expression in each of the three regions of the native human lens; anterior epithelia, equatorial region epithelia and fibers. The x-axis represents the gene of interest/18S expression calculated as mean±SEM. In individual native lens regions significance at p≤0.05 (*) was determined (ANOVA with Tukey's post-hoc analysis) in that region versus the other two regions of each lens.

To provide a measure of the data for the comparison of *ADAM*/*ADAMTS* gene expression between the three lens regions the data were normalized relative to the level of an endogenous control gene, 18S rRNA message, in each sample (Figure 2). There were no statistical differences in gene expression of *ADAM-9*, *-10*, and *-17* genes between the regions examined (Figure 2A). However, regional differences were observed with *ADAM-8* and *-15* and a similar distribution pattern for both was seen (Figure 2A), such that anterior epithelia>equatorial region>fibers; a reciprocal pattern was found for *ADAM-19* (i.e., where most expression was detected in the fibers).

*ADAMTS-1* and *-2* gene expression was highest in the equatorial region, while the anterior epithelia and fibers had low expression of *ADAMTS-1* and *-2* genes (Figure 2B). This pattern of regional expression was significantly different for *ADAMTS-1* only, however it should be noted that of three donors analyzed the pattern was consistent. In the case of *ADAMTS-3* and *-14* there was no significant difference in gene expression across the three regions (Figure 2B).

### *ADAM*/*ADAMTS* gene expression determined by C_T_ analysis in the wounded human lens

**Figure 3:**
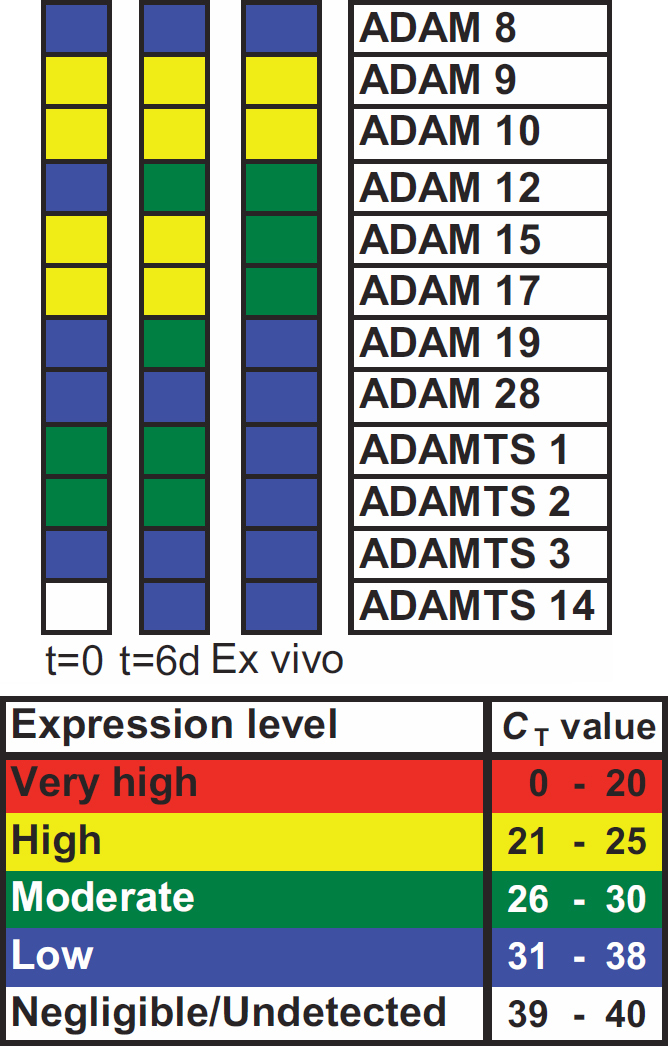
*ADAM*/*ADAMTS* gene expression in response to mechanical trauma. Gene expression profiles were classified in CBs; at t=0 following in vitro sham cataract operation (left panel), at t=6d, following short-term culture (central panel) and ex vivo after cataract surgery (right panel). The cycle threshold number (C_T_) was used to classify gene expression as either very high (C_T_≤20), high (C_T_=21–25), moderate (C_T_=26–30), low (C_T_=31–38), or negligible to undetected (C_T_=39–40). Donor information; category (t=0, 6d, ex vivo), sex (F; female, M; male), age and ophthalmic history. t=0: F, 74, none; M, 83, none; M, 81, none. t=6d; M, 76, none; F, 76, none; M, 69, no info. Ex vivo; M, 75, bilateral cataract surgery 2001; F, 76, no info; M, 70, no info.

In the qualitative analysis of the raw data cycle threshold (C_T_) value, before 18S normalization (Figure 3), *ADAM-12*, *-19*, and *ADAMTS-14* showed a positive shift in gene expression during short-term culture (t=6d) relative to t=0. The expression of *ADAM-12* and *-19* increased from low to moderate and *ADAMTS-14*, which was negligible/undetected before culture, increased to a low expression level. The classification of the remaining *ADAM*/*ADAMTS* genes expressed (*ADAM-8*, *-9*, *-10*, *-15*, *-17*, *-28*, *ADAMTS-1*, *-2*, and *-3*) did not change with short-term culture relative to t=0.

In ex vivo capsular bags, the gene expression of all *ADAM*/*ADAMTS* candidates was detectable. The majority of *ADAM*/*ADAMTS* were detected at a low level with the exception of *ADAM-9* and *-10*, which were present at a high level and *ADAM-12*, *-15*, and *-17* were moderately expressed.

Comparison of expression in ex vivo capsular bags to t=0 showed that *ADAM-8*, *-9*, *-10*, *-19*, *-28*, and *ADAMTS-3* remained the same. Several the *ADAM*/*ADAMTS* genes showed an expression differential between ex vivo and t=0 capsular bags; an increase in *ADAM-12* (low elevated to moderate) and *ADAMTS-14* (negligible/undetected elevated to low) and a decrease in *ADAM-15*, *-17* (high reduced to moderate), and *ADAMTS-1* and *-2* (moderate reduced to low) was detected.

### *ADAM*/*ADAMTS* gene expression in the wounded human lens determined using a relative standard curve

**Figure 4:**
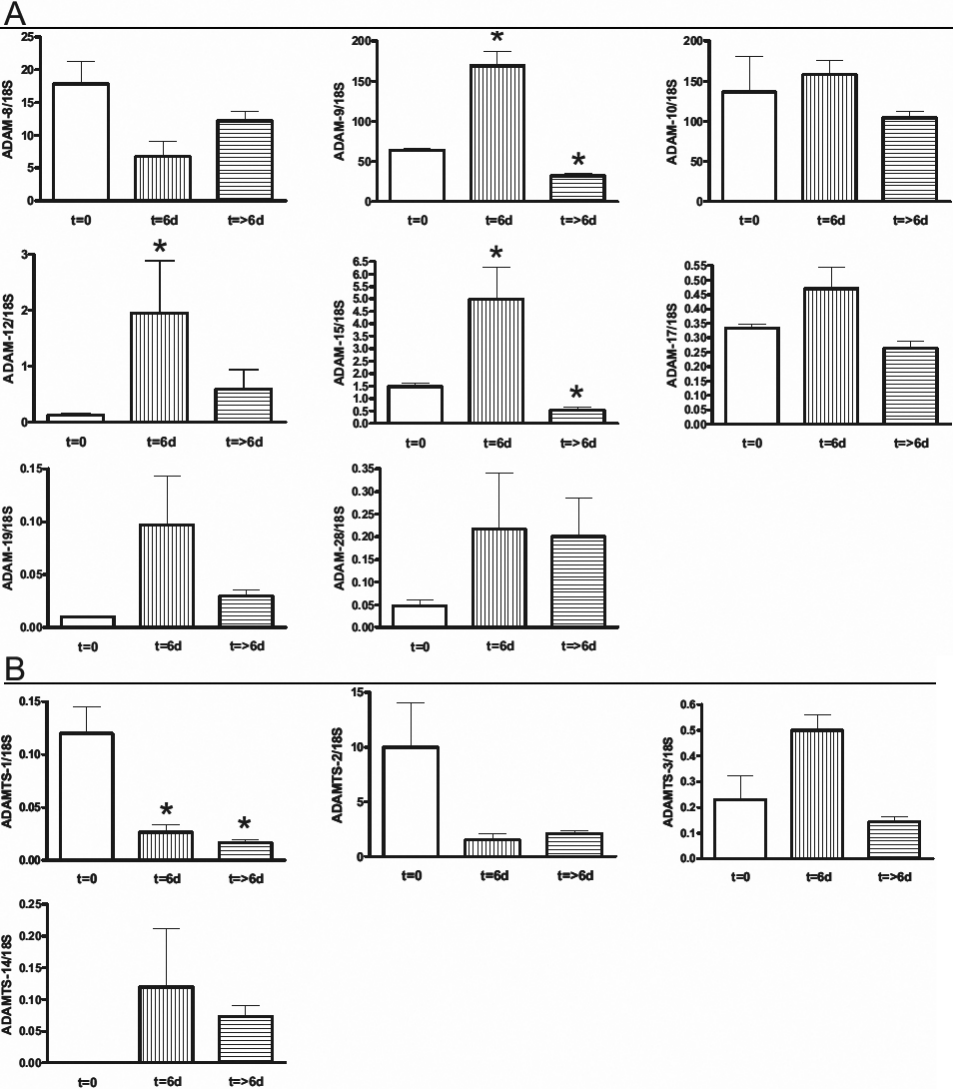
Quantitative assessment of *ADAM*/*ADAMTS* gene expression in response to mechanical trauma. **A**: *ADAM* and **B**: *ADAMTS* gene expression analysis in human capsular bags; at t=0 following in vitro sham cataract operation, at t=6d, following short-term culture and ex vivo after cataract surgery. The y-axis represents the gene of interest/18S expression calculated as mean±SEM * indicates a ignificant difference in gene expression between groups at p≤0.05 determined by ANOVA with Tukey's post-hoc analysis.

The quantitative analysis of *ADAM*/*ADAMTS* gene expression, in which data were expressed relative to 18S ribosomal control, is shown in Figure 4. Following surgical trauma, a significant increase in expression of *ADAM-9*, *-15*, and *ADAMTS-3*/*ADAMTS* (*ADAM-9*, *-12*, *-15*, *-19*, *-28*, *ADAMTS-3*, and *-14*) was detected in t=6d capsular bags (Figure 4A,B). While only *ADAM-9*, *-15*, and *ADAMTS-3* at t=6d reached a statistically significant difference from t=0. After short-term culture, the expression of *ADAM-10*, *-12*, *-19*, *-28*, *ADAMTS-2*, and *-14* remained unchanged from t=0 (Figure 4A,B). A significant reduction in the expression was detected for *ADAMTS-1* (Figure 4B).

In comparison with t=0 capsular bags, the gene expression of the ex vivo samples was largely unchanged, except for *ADAM-28* and *ADAMTS-14* that increased and *ADAMTS-1* and *-2* that decreased (Figure 4A,B). Notably, *ADAM-9* gene expression was significantly different from t=0 and was reduced by 50.2%. The change in *ADAMTS-1* was the only statistically significant gene expression differential detected.

## Discussion

### Adamalysin gene expression in native lens regions

The ADAMs are known to modulate cell-cell and cell-matrix interaction with cell surface adhesion molecules and integrins through various mechanisms including protein ectodomain shedding and the adhesive qualities of the disintegrin and Cys-rich domains. In the mouse lens, the particular importance of integrin adhesion molecules is demonstrated by the dramatic lens microphthalmia that occurs in the adult β1 integrin knock out [23]. Human ADAM-15 in particular contains an RGD integrin-binding motif [24] and others alternatively contain sequences such as ECD or DCD [2,25]. The ADAM-15 RGD motif is involved in adherence to αvβ3 and α5β1 integrins [26,27]. However, ADAM-15 can additionally bind α9β1 integrins in an RGD-independent mechanism [28], which indicates the adhesive potential for ADAMs lacking an RGD motif. Cell contact and adhesion are likely to be functions common to all lens cells and may account for the high and consistent expression of particular ADAM genes such as ADAM-9, −10, −15 and −17 in each of the native human lens regions determined by the present study. ADAM-9 is a potential ligand for α6β1 integrin [29] and the α6 and β1 integrin subunit genes are expressed by the human lens epithelia-derived cell line FHL-124 [30]. Monoclonal antibodies have detected the β1 subunit expressed by lens epithelial cells of capsulorhexis specimens removed at surgery [31] and it is detected at the fiber cell-capsule interface [32]. These observations indicate a potential adhesive role for ADAM-9 in the lens epithelia and during fiber differentiation.

In keeping with the theme of cell contact and adhesion, in non-ocular tissues ADAM-10 is implicated in the normal turnover of cadherin adhesion molecules [33] and in the shedding of cell surface adhesion proteins such as CD44v6 [10]. Both of these examples are mediated by protein ectodomain cleavage and are relevant to the maintenance of native lens compartments. Roles in adhesion via integrin interactions are similarly reported for ADAM-15 and −17 and may be involved in maintaining the quiescence of the native lens epithelia and controlling migration in lens cells. For example, the quantitative data presented in Figure 2 show that ADAM-15 was significantly expressed by the quiescent anterior lens epithelia. In airway smooth muscle cells a recombinant ADAM-15 disintegrin domain construct prevented PDGF-induced matrix adhesion and migration through the β1 integrin [34]. Also migration of human lens epithelial cells induced in culture can be prevented with an interacting anti-β1 monoclonal antibody [31]. Therefore, an ADAM-15-β1 integrin interaction may prevent cell migration of the anterior epithelia. Moreover, in support of an inhibitory interaction the integrin binding characteristics of ADAM-15 can prevent platelet aggregation [35].

Consistently high *ADAM-17* gene expression was detected in all native lens regions. In vitro, ADAM-17-mediated adhesion occurs between the disintegrin region (in an RGD peptide-sensitive interaction) and α5β1 integrin localized at focal adhesions in sparsely seeded HeLa cell cultures and cell-cell junctions in confluent cultures [36]. A further in vitro study demonstrated that ADAM-17 inhibits migration through α5β1 integrin but not α4β1 [37]. Taken together, these studies suggest that the expression of differing integrin receptor subtypes target or modulate ADAM activity to support roles in cellular adhesion and migration, and in the lens could play a role in tissue integrity in the samples investigated.

Of the 4 ADAMTS family members studied, ADAMTS-1 and ADAMTS-2 were the most abundant and resided largely in the equatorial region. At the lens equator multiple processes are occurring, including cell division and migration and differentiation. These processes continue to occur throughout life and contribute to the persistent, but linear increase of human lens growth with age [38-40]. Moreover, injury to the lens, such as a cataract surgery can provoke enhanced levels of cell proliferation and migration [40,41]. This increased activity occurs in all ages, but is greater in the young [41,42]. The promotion of growth responses following injury contribute greatly to posterior capsule opacification and explain why it is a major healthcare problem affecting millions, of largely elderly patients [4]. In other tissues ADAMTS-1 has been associated with migration and proliferation. For example, ADAMTS-1 can be detected in immortalized human corneal fibroblasts [11] and a binding of a co-factor fibulin-1 promotes aggrecan cleavage [43], which can regulate migration [11]. It is therefore feasible that ADAMTS-1 facilitates cell division, migration and differentiation in the lens.

### Adamalysin gene expression in the wounded lens

The short-term lens wounding stimulus led to a significant increase in *ADAM-9* and *-15* gene expression. Among the genes that have been associated with the wounding response are the epidermal growth factor receptors (EGFRs). The EGFRs are transactivated by G-protein-coupled-receptors (GPCRs), in a mechanism that involves metalloproteinase-dependent shedding of EGFR ligands, subsequent EGFR activation and the initiation of downstream cell signaling [44]. For example in the dermis, EGFR ligands including heparin binding-EGF (HB-EGF), are proteolytically shed from the cell surface in response to injury and play leading roles in wound healing, including increasing cell migration [45,46]. In the anterior segment, corneal wounding stimulates release of the GPCR agonist lysophosphatidic acid (LPA), which accelerates wound healing i.e., cell migration and wound closure through shedding of HB-EGF and EGFR transactivation [47]. Thus, ADAM-mediated proteolysis plays an important part in releasing proteins from the cell surface to modulate their bioavailability to change cell behavior.

In the lens, there is a lack of research to substantiate the role of the ADAMs in EGFR ligand shedding and this is an area that needs addressing. However, the upregulation of the ADAM proteases has been previously documented in activated cells of other tissues [48]. ADAM-9 is known to shed HB-EGF from the cell surface when the cytoplasmic domain is bound by protein kinase C delta [49]. ADAM-15 is implicated in EGFR ligand shedding, possibly through EGFR transactivation [50,51] and catalyzes the shedding of a soluble E-cadherin fragment that is increased during the progression of breast cancer [52]. EGFR ligands including HB-EGF are expressed by the lens epithelial cell line, FHL 124, which is suggested to better represent a wounded lens cell line (due to the expression of genes usually associated with vigorously growing or wounded lens cells) [30]. Moreover, despite the lack of information regarding ADAM sheddase activity against EGFR ligands in the lens, the role of the EGF/R stimulation pathway in lens cells has been under investigation for some time and the presence of EGF and EGFR is documented in human lens epithelial cells [53-56]. The EGFR immunolocalizes to both the quiescent anterior and dividing equatorial epithelial cells of the native human lens and EGFR activation by EGF is strongest at the equator [55]. EGFRs are similarly detectable in activated lens epithelial cells that are proliferating and migrating following sham surgical injury and EGFR blockade can decrease the rate of this lens epithelial cell growth [55]. In other studies EGF treatment has been shown to promote the migration of human lens epithelial cells [57]. One can therefore conclude that ADAM-9 and −15, which are elevated following surgery, may potentially be involved in the processes that propagate a wound response in lens cells, which may ultimately lead to the development of PCO secondary to cataract surgery.

The data generated from long-term samples (ex vivo) indicate a reduction in *ADAM-9* gene expression following surgical trauma. Interestingly, a down-regulation in *ADAM-9* gene expression has been associated with human anterior polar cataract or ASC [43]. The α6β1 integrins are suggested to be potential ADAM-9 receptors [29]. β1 integrins are expressed by lens cells [30-32] and TGF-β1 down-regulates *ADAM-9* and the α6 integrin subunit [13,58], which highlights that a down-regulation of *ADAM-9* could be involved in a loss of cell-cell contact during the pathogenesis of anterior sub-capsular cataract (ASC) and potentially also PCO, as they are phenotypically similar [59,60].

Importantly, ADAM-10 is one of the ADAMs that is highly expressed in the long-term wounding samples. In aberrantly activated lens epithelial cells ADAM-10 is likely to conserve its proteolytic functions for the cell proliferation and migration that is seen in the development of PCO. In other tissues, ADAM-10 is associated with aberrant cadherin processing, which is thought to cause downstream functional alterations in cell-cell adhesion and β-catenin signaling in the pathogenesis of disease states such as cancer and Alzheimer disease [61,62]. The destabilization of cell-cell junctions has also been associated with cataract in induced animal models via TGFβ [63] and stress [64]. TGFβ induced E-cadherin shedding in an ex vivo rat cataract model was attributed to members of the matrix mettalloproteinases [63]. However, ADAM-10 could be equally responsible for E-cadherin shedding and may be involved in the pathogenesis of ASC and PCO.

Of the ADAMTS family members only ADAMTS-1 expression was significantly changed following injury; however ADAMTS-2 showed a similar pattern. Following injury, expression was dramatically reduced; suggesting ADAMTS-1 is not directly driving a wound-healing response. Wound-healing responses are associated with enhanced migration and division and this is typical of events following cataract surgery [4]. Interestingly, it has been observed that hormonally stimulated osteoblasts upregulate *ADAMTS-1* expression on collagen coated dishes, which is associated with reduced migration [65]. Therefore, if *ADAMTS-1* expression suppresses migration then the observed reduction in lens cells following injury is likely to promote migration. This concept is supported by other studies, which report that low concentrations of ADAMTS-1 were seen to stimulate fibroblast migration in healing skin wounds, whereas high concentrations inhibited migration through sequestration of fibroblast growth factor [66].

In conclusion, we have provided a robust analysis of *ADAM*/*ADAMTS* gene expression in the normal and wounded human lens and have described their potential functional implications. An important unexplored theme is the ability of TGFβ to regulate *ADAM*/*ADAMTS* expression because this may modulate cellular adhesion for roles in the migration and proliferation of transdifferentiated lens cells; lens epithelial cell transdifferentiation is promoted by TGFβ and involved in PCO development [4,67]. The relationship between, ADAM/ADAMTS, TGFβ, and PCO therefore warrants further investigation in the future.

## References

[1] Edwards DR Handsley MM Pennington CJ The ADAM metalloproteinases. Mol Aspects Med 2008 29 258 89 18762209 10.1016/j.mam.2008.08.001 PMC7112278

[2] Blobel CP ADAMs: key components in EGFR signalling and development. Nat Rev Mol Cell Biol 2005 6 32 43 15688065 10.1038/nrm1548

[3] Porter S Clark IM Kevorkian L Edwards DR The ADAMTS metalloproteinases. Biochem J 2005 386 15 27 15554875 10.1042/BJ20040424 PMC1134762

[4] Wormstone IM Wang L Liu CS Posterior capsule opacification. Exp Eye Res 2009 88 257 69 19013456 10.1016/j.exer.2008.10.016

[5] White JM ADAMs: modulators of cell-cell and cell-matrix interactions. Curr Opin Cell Biol 2003 15 598 606 14519395 10.1016/j.ceb.2003.08.001

[6] Seals DF Courtneidge SA The ADAMs family of metalloproteases: multidomain proteins with multiple functions. Genes Dev 2003 17 7 30 12514095 10.1101/gad.1039703

[7] Tousseyn T Jorissen E Reiss K Hartmann D (Make) stick and cut loose–disintegrin metalloproteases in development and disease. Birth Defects Res C Embryo Today 2006 78 24 46 16622847 10.1002/bdrc.20066

[8] Jones GC Riley GP ADAMTS proteinases: a multi-domain, multi-functional family with roles in extracellular matrix turnover and arthritis. Arthritis Res Ther 2005 7 160 9 15987500 10.1186/ar1783 PMC1175049

[9] Kuno K Kanada N Nakashima E Fujiki F Ichimura F Matsushima K Molecular cloning of a gene encoding a new type of metalloproteinase-disintegrin family protein with thrombospondin motifs as an inflammation associated gene. J Biol Chem 1997 272 556 62 8995297 10.1074/jbc.272.1.556

[10] Huh MI Lee YM Seo SK Kang BS Chang Y Lee YS Fini ME Kang SS Jung JC Roles of MMP/TIMP in regulating matrix swelling and cell migration during chick corneal development. J Cell Biochem 2007 101 1222 37 17295208 10.1002/jcb.21246

[11] Ducros E Berthaut A Mirshahi P Lemarchand S Soria J Legeais JM Mirshahi M Expression of extracellular matrix proteins fibulin-1 and fibulin-2 by human corneal fibroblasts. Curr Eye Res 2007 32 481 90 17612964 10.1080/02713680701411269

[12] Watabe-Uchida M Masuda A Shimada N Endo M Shimamura K Yasuda K Sehara-Fujisawa A Novel metalloprotease-disintegrin, meltrin epsilon (ADAM35), expressed in epithelial tissues during chick embryogenesis. Dev Dyn 2004 230 557 68 15188440 10.1002/dvdy.20052

[13] Lim JM Lee JH Wee WR Joo CK Downregulated expression of ADAM9 in anterior polar cataracts. J Cataract Refract Surg 2002 28 697 702 11955914 10.1016/s0886-3350(01)01236-6

[14] Dagoneau N Benoist-Lasselin C Huber C Faivre L Megarbane A Alswaid A Dollfus H Alembik Y Munnich A Legeai-Mallet L Cormier-Daire V ADAMTS10 mutations in autosomal recessive Weill-Marchesani syndrome. Am J Hum Genet 2004 75 801 6 15368195 10.1086/425231 PMC1182109

[15] Ovodenko B Rostagno A Neubert TA Shetty V Thomas S Yang A Liebmann J Ghiso J Ritch R Proteomic analysis of exfoliation deposits. Invest Ophthalmol Vis Sci 2007 48 1447 57 17389470 10.1167/iovs.06-0411

[16] Ritch R Schlotzer-Schrehardt U Exfoliation syndrome. Surv Ophthalmol 2001 45 265 315 11166342 10.1016/s0039-6257(00)00196-x

[17] Hodgkinson LM Duncan G Wang L Pennington CJ Edwards DR Wormstone IM MMP and TIMP expression in quiescent, dividing, and differentiating human lens cells. Invest Ophthalmol Vis Sci 2007 48 4192 9 17724206 10.1167/iovs.06-1371

[18] Liu CS Wormstone IM Duncan G Marcantonio JM Webb SF Davies PD A study of human lens cell growth in vitro. A model for posterior capsule opacification. Invest Ophthalmol Vis Sci 1996 37 906 14 8603875

[19] Koshy PJ Lundy CJ Rowan AD Porter S Edwards DR Hogan A Clark IM Cawston TE The modulation of matrix metalloproteinase and ADAM gene expression in human chondrocytes by interleukin-1 and oncostatin M: a time-course study using real-time quantitative reverse transcription-polymerase chain reaction. Arthritis Rheum 2002 46 961 7 11953973 10.1002/art.10212

[20] Jones GC Corps AN Pennington CJ Clark IM Edwards DR Bradley MM Hazleman BL Riley GP Expression profiling of metalloproteinases and tissue inhibitors of metalloproteinases in normal and degenerate human achilles tendon. Arthritis Rheum 2006 54 832 42 16508964 10.1002/art.21672

[21] Porter S Scott SD Sassoon EM Williams MR Jones JL Girling AC Ball RY Edwards DR Dysregulated expression of adamalysin-thrombospondin genes in human breast carcinoma. Clin Cancer Res 2004 10 2429 40 15073121 10.1158/1078-0432.ccr-0398-3

[22] Nuttall RK Pennington CJ Taplin J Wheal A Yong VW Forsyth PA Edwards DR Elevated membrane-type matrix metalloproteinases in gliomas revealed by profiling proteases and inhibitors in human cancer cells. Mol Cancer Res 2003 1 333 45 12651907

[23] Simirskii VN Wang Y Duncan MK Conditional deletion of beta1-integrin from the developing lens leads to loss of the lens epithelial phenotype. Dev Biol 2007 306 658 68 17493607 10.1016/j.ydbio.2007.04.004 PMC1950782

[24] Niewiarowski S McLane MA Kloczewiak M Stewart GJ Disintegrins and other naturally occurring antagonists of platelet fibrinogen receptors. Semin Hematol 1994 31 289 300 7831574

[25] Kamiguti AS Zuzel M Theakston RD Snake venom metalloproteinases and disintegrins: interactions with cells. Braz J Med Biol Res 1998 31 853 62 9698750 10.1590/s0100-879x1998000700001

[26] Nath D Slocombe PM Stephens PE Warn A Hutchinson GR Yamada KM Docherty AJ Murphy G Interaction of metargidin (ADAM-15) with alphavbeta3 and alpha5beta1 integrins on different haemopoietic cells. J Cell Sci 1999 112 579 87 9914169 10.1242/jcs.112.4.579

[27] Zhang XP Kamata T Yokoyama K Puzon-McLaughlin W Takada Y Specific interaction of the recombinant disintegrin-like domain of MDC-15 (metargidin, ADAM-15) with integrin alphavbeta3. J Biol Chem 1998 273 7345 50 9516430 10.1074/jbc.273.13.7345

[28] Eto K Puzon-McLaughlin W Sheppard D Sehara-Fujisawa A Zhang XP Takada Y RGD-independent binding of integrin alpha9beta1 to the ADAM-12 and −15 disintegrin domains mediates cell-cell interaction. J Biol Chem 2000 275 34922 30 10944520 10.1074/jbc.M001953200

[29] Wederell ED de Iongh RU Extracellular matrix and integrin signaling in lens development and cataract. Semin Cell Dev Biol 2006 17 759 76 17134921 10.1016/j.semcdb.2006.10.006

[30] Dawes LJ Elliott RM Reddan JR Wormstone YM Wormstone IM Oligonucleotide microarray analysis of human lens epithelial cells: TGFbeta regulated gene expression. Mol Vis 2007 13 1181 97 17679943

[31] Nishi O Nishi K Akaishi T Shirasawa E Detection of cell adhesion molecules in lens epithelial cells of human cataracts. Invest Ophthalmol Vis Sci 1997 38 579 85 9071210

[32] Walker JL Menko AS alpha6 Integrin is regulated with lens cell differentiation by linkage to the cytoskeleton and isoform switching. Dev Biol 1999 210 497 511 10357906 10.1006/dbio.1999.9277

[33] Reiss K Maretzky T Ludwig A Tousseyn T de Strooper B Hartmann D Saftig P ADAM10 cleavage of N-cadherin and regulation of cell-cell adhesion and beta-catenin nuclear signalling. EMBO J 2005 24 742 52 15692570 10.1038/sj.emboj.7600548 PMC549617

[34] Lu D Xie S Sukkar MB Lu X Scully MF Chung KF Inhibition of airway smooth muscle adhesion and migration by the disintegrin domain of ADAM-15. Am J Respir Cell Mol Biol 2007 37 494 500 17575078 10.1165/rcmb.2006-0364OC

[35] Lu D Chung KF Xia M Lu X Scully M Kakkar V Integrin binding characteristics of the disintegrin-like domain of ADAM-15. Thromb Haemost 2006 96 642 51 17080222

[36] Bax DV Messent AJ Tart J van Hoang M Kott J Maciewicz RA Humphries MJ Integrin alpha5beta1 and ADAM-17 interact in vitro and co-localize in migrating HeLa cells. J Biol Chem 2004 279 22377 86 14970227 10.1074/jbc.M400180200

[37] Huang J Bridges LC White JM Selective modulation of integrin-mediated cell migration by distinct ADAM family members. Mol Biol Cell 2005 16 4982 91 16079176 10.1091/mbc.E05-03-0258 PMC1237097

[38] Augusteyn RC On the growth and internal structure of the human lens. Exp Eye Res 2010 90 643 54 20171212 10.1016/j.exer.2010.01.013 PMC2871961

[39] Augusteyn RC Growth of the human eye lens. Mol Vis 2007 13 252 7 17356512 PMC2633484

[40] Rakic JM Galand A Vrensen GF Separation of fibres from the capsule enhances mitotic activity of human lens epithelium. Exp Eye Res 1997 64 67 72 9093022 10.1006/exer.1996.0179

[41] Wormstone IM Liu CS Rakic JM Marcantonio JM Vrensen GF Duncan G Human lens epithelial cell proliferation in a protein-free medium. Invest Ophthalmol Vis Sci 1997 38 396 404 9040473

[42] Moisseiev J Bartov E Schochat A Blumenthal M Long-term study of the prevalence of capsular opacification following extracapsular cataract extraction. J Cataract Refract Surg 1989 15 531 3 2810087 10.1016/s0886-3350(89)80110-5

[43] Lee NV Rodriguez-Manzaneque JC Thai SN Twal WO Luque A Lyons KM Argraves WS Iruela-Arispe ML Fibulin-1 acts as a cofactor for the matrix metalloprotease ADAMTS-1. J Biol Chem 2005 280 34796 804 16061471 10.1074/jbc.M506980200

[44] Higashiyama S Iwabuki H Morimoto C Hieda M Inoue H Matsushita N Membrane-anchored growth factors, the epidermal growth factor family: beyond receptor ligands. Cancer Sci 2008 99 214 20 18271917 10.1111/j.1349-7006.2007.00676.x PMC11158050

[45] Shirakata Y Kimura R Nanba D Iwamoto R Tokumaru S Morimoto C Yokota K Nakamura M Sayama K Mekada E Higashiyama S Hashimoto K Heparin-binding EGF-like growth factor accelerates keratinocyte migration and skin wound healing. J Cell Sci 2005 118 2363 70 15923649 10.1242/jcs.02346

[46] Tokumaru S Higashiyama S Endo T Nakagawa T Miyagawa JI Yamamori K Hanakawa Y Ohmoto H Yoshino K Shirakata Y Matsuzawa Y Hashimoto K Taniguchi N Ectodomain shedding of epidermal growth factor receptor ligands is required for keratinocyte migration in cutaneous wound healing. J Cell Biol 2000 151 209 20 11038170 10.1083/jcb.151.2.209 PMC2192647

[47] Xu KP Yin J Yu FS Lysophosphatidic acid promoting corneal epithelial wound healing by transactivation of epidermal growth factor receptor. Invest Ophthalmol Vis Sci 2007 48 636 43 17251460 10.1167/iovs.06-0203 PMC2665794

[48] Carl-McGrath S Lendeckel U Ebert M Roessner A Rocken C The disintegrin-metalloproteinases ADAM9, ADAM12, and ADAM15 are upregulated in gastric cancer. Int J Oncol 2005 26 17 24 15586220

[49] Izumi Y Hirata M Hasuwa H Iwamoto R Umata T Miyado K Tamai Y Kurisaki T Sehara-Fujisawa A Ohno S Mekada E A metalloprotease-disintegrin, MDC9/meltrin-gamma/ADAM9 and PKCdelta are involved in TPA-induced ectodomain shedding of membrane-anchored heparin-binding EGF-like growth factor. EMBO J 1998 17 7260 72 9857183 10.1093/emboj/17.24.7260 PMC1171072

[50] Hart S Fischer OM Prenzel N Zwick-Wallasch E Schneider M Hennighausen L Ullrich A GPCR-induced migration of breast carcinoma cells depends on both EGFR signal transactivation and EGFR-independent pathways. Biol Chem 2005 386 845 55 16164409 10.1515/BC.2005.099

[51] Schäfer B Marg B Gschwind A Ullrich A Distinct ADAM metalloproteinases regulate G protein-coupled receptor-induced cell proliferation and survival. J Biol Chem 2004 279 47929 38 15337756 10.1074/jbc.M400129200

[52] Najy AJ Day KC Day ML The ectodomain shedding of E-cadherin by ADAM15 supports ErbB receptor activation. J Biol Chem 2008 283 18393 401 18434311 10.1074/jbc.M801329200 PMC2440598

[53] Bhuyan DK Reddy PG Bhuyan KC Growth factor receptor gene and protein expressions in the human lens. Mech Ageing Dev 2000 113 205 18 10714939 10.1016/s0047-6374(99)00111-6

[54] Lee EH Joo CK Role of transforming growth factor-beta in transdifferentiation and fibrosis of lens epithelial cells. Invest Ophthalmol Vis Sci 1999 40 2025 32 10440257

[55] Maidment JM Duncan G Tamiya S Collison DJ Wang L Wormstone IM Regional differences in tyrosine kinase receptor signaling components determine differential growth patterns in the human lens. Invest Ophthalmol Vis Sci 2004 45 1427 35 15111598 10.1167/iovs.03-1187

[56] Majima K Kojima Y Ouhashi F Cell biological analysis with respect to cause of fibrous opacification of the anterior capsule after cataract extraction. Ophthalmologica 1998 212 364 8 9787225 10.1159/000027369

[57] Wang J Li X Zhang X Sun H Yuan J Effect of epidermal growth factor on proliferation of lens epithelial cells. Zhonghua Yan Ke Za Zhi 1999 35 283 6 11835824

[58] Lim JM Kim JA Lee JH Joo CK Downregulated expression of integrin alpha6 by transforming growth factor-beta(1) on lens epithelial cells in vitro. Biochem Biophys Res Commun 2001 284 33 41 11374867 10.1006/bbrc.2001.4942

[59] Hatae T Ishibashi T Yoshitomi F Shibata Y Immunocytochemistry of types I–IV collagen in human anterior subcapsular cataracts. Graefes Arch Clin Exp Ophthalmol 1993 231 586 90 8224934 10.1007/BF00936523

[60] Schmitt-Gräff A Pau H Spahr R Piper HM Skalli O Gabbiani G Appearance of alpha-smooth muscle actin in human eye lens cells of anterior capsular cataract and in cultured bovine lens-forming cells. Differentiation 1990 43 115 22 2373284 10.1111/j.1432-0436.1990.tb00437.x

[61] Klucky B Mueller R Vogt I Teurich S Hartenstein B Breuhahn K Flechtenmacher C Angel P Hess J Kallikrein 6 induces E-cadherin shedding and promotes cell proliferation, migration, and invasion. Cancer Res 2007 67 8198 206 17804733 10.1158/0008-5472.CAN-07-0607

[62] Uemura K Kihara T Kuzuya A Okawa K Nishimoto T Ninomiya H Sugimoto H Kinoshita A Shimohama S Characterization of sequential N-cadherin cleavage by ADAM10 and PS1. Neurosci Lett 2006 402 278 83 16687212 10.1016/j.neulet.2006.04.018

[63] Dwivedi DJ Pino G Banh A Nathu Z Howchin D Margetts P Sivak JG West-Mays JA Matrix metalloproteinase inhibitors suppress transforming growth factor-beta-induced subcapsular cataract formation. Am J Pathol 2006 168 69 79 16400010 10.2353/ajpath.2006.041089 PMC1592675

[64] Zhou J Leonard M Van Bockstaele E Menko AS Mechanism of Src kinase induction of cortical cataract following exposure to stress: destabilization of cell-cell junctions. Mol Vis 2007 13 1298 310 17679932

[65] Rehn AP Birch MA Karlstrom E Wendel M Lind T ADAMTS-1 increases the three-dimensional growth of osteoblasts through type I collagen processing. Bone 2007 41 231 8 17560840 10.1016/j.bone.2007.04.187

[66] Krampert M Kuenzle S Thai SN Lee N Iruela-Arispe ML Werner S ADAMTS1 proteinase is up-regulated in wounded skin and regulates migration of fibroblasts and endothelial cells. J Biol Chem 2005 280 23844 52 15843381 10.1074/jbc.M412212200

[67] Wormstone IM Posterior capsule opacification: a cell biological perspective. Exp Eye Res 2002 74 337 47 12014915 10.1006/exer.2001.1153

